# Domestic abuse among female doctors: thematic analysis of qualitative interviews in the UK

**DOI:** 10.3399/BJGP.2020.0795

**Published:** 2021-02-09

**Authors:** Emily Donovan, Miriam Santer, Sara Morgan, Gavin Daker-White, Merlin Willcox

**Affiliations:** Population Sciences and Medical Education, University of Southampton, Southampton.; Population Sciences and Medical Education, University of Southampton, Southampton.; Population Sciences and Medical Education, University of Southampton, Southampton.; Centre for Primary Care, University of Manchester, Manchester.; Population Sciences and Medical Education, University of Southampton, Southampton.

**Keywords:** abused woman, domestic violence, interpersonal violence, peer support, qualitative research, spouse abuse, women physicians

## Abstract

**Background:**

Doctors can be victim-survivors of domestic abuse (DA), but how this impacts their work and wellbeing, and whether they face barriers to seeking help is not well understood.

**Aim:**

To understand single doctor mothers’ lived experience of DA, barriers to seeking help, and impact on their work.

**Design and setting:**

Individual qualitative interviews with female doctors in the UK who had left an abusive relationship. Interviews were conducted between August 2019 and March 2020.

**Method:**

Participants were invited via a closed online forum for female doctors who are single parents. In total, 114 females expressed interest. In-depth semi-structured telephone interviews were audiorecorded and transcribed. Transcripts were uploaded to NVivo and analysed using inductive thematic analysis.

**Results:**

A total of 21 participants were interviewed. The internalised stigma of DA affected participants’ sense of identity and belonging as a doctor, causing social and professional isolation. Many participants felt that the acute stress of DA had an impact on their work, yet often felt unable to take time off. Barriers to seeking help included lack of confidentiality, especially where the abusive partner was also a doctor (sometimes accusing the victim-survivor of mental illness or threatening to report them to the General Medical Council). Participants found peer support helpful, as well as consulting health professionals who were empathic towards them. After they had left the abusive relationship victim-survivors felt better equipped to support patients going through DA.

**Conclusion:**

Domestic abuse impacts on the work and wellbeing of female doctors, who face unique barriers to help seeking and reporting DA. An online peer support group can help to break the sense of isolation, but specialised confidential support services are also required to help doctors experiencing DA.

## INTRODUCTION

The current government definition of domestic abuse (DA) is as follows:

*‘Any incident or pattern of incidents of controlling, coercive, threatening behaviour, violence or abuse between those aged 16 or over who are, or have been, intimate partners or family members regardless of gender or sexuality. It can encompass, but is not limited to: psychological, physical, sexual, financial and emotional abuse.’* ^1^

Victim-survivors of DA suffer with physical and mental health consequences that can be long lasting.^2^ Approximately 7.5% of females in England and Wales experienced DA in the year ending March 2019 with females around twice as likely to experience DA as males.^1^ DA is ‘ *a serious public health issue that crosses geographical, demographic and socio-economic boundaries.*’^3^ Two females are murdered each week by their intimate partner in England and Wales.^4^

In the UK, female nurses are three times more likely to be a victim of DA than females in the general public,^5^ and in Australia female health professionals report a higher prevalence of DA compared with the general population.^6^ The Royal College of Midwives has reported insufficient workplace support for midwife victim-survivors.^7^ A literature review of interpersonal violence and physician victim-survivors^8^ highlighted the ‘extreme stigma’ among physicians that may prevent them from seeking help, concluding that more research is needed in this area. Further, previous qualitative research has focused on a doctor’s role in supporting victim-survivors of DA^1^^,^^2^^,^^9^^,^^10^ reinforcing the stereotype that the doctor helps others experiencing DA rather than being a victim-survivor themselves.

An evaluation of an Independent Domestic Violence Advisor (IDVA) hospital programme found that staff sought advice for themselves personally even though the IDVA was there to support patients.^11^ Evaluation of DA training for healthcare professionals (HPs) found ‘ *an unintended consequence was the disclosure of DA among staff’*, highlighting a need for support mechanisms for staff.^12^ It is important to understand doctors’ experience of DA to help employers, colleagues, and HPs respond positively, and to understand how aspects of working as a doctor may increase the risk of becoming a victim-survivor. New UK legislation, in the form of the Domestic Abuse Bill 2019, recognises that employers have a important role in supporting victim-survivors of DA.^13^

The present qualitative interview study was undertaken in order to explore doctor mothers’ experiences of DA, barriers to seeking help, and the impact on their work. While the experiences of other doctors around DA would also be important for further study, the present study’s focus is on doctor mothers because this is a group to which the authors had access since the first author was a member of the online forum and a single parent female doctor. The researchers did not focus on additional issues that victim-survivors face when they have children as this is well documented elsewhere.^14^

**Table 1:** How this fits in

Previous qualitative research has focused on a doctor’s role in supporting victim-survivors of domestic abuse (DA) reinforcing the stereotype in which the doctor helps others experiencing DA rather than being a victim-survivor themselves. This study explored single doctor mothers’ lived experience of DA and identified several unique barriers to seeking help. Healthcare professionals need to be aware of these in order to better support patients who are medical doctors.

## METHOD

Participants were recruited from a private online forum for single parents who are all female doctors, and are single through choice, bereavement, or breakdown of a relationship. The study’s first author posted a message in May 2019 asking females who considered themselves to have been a victim-survivor of DA whether they would consider participating in a telephone interview study. Undertaking the interviews by telephone carried the advantage of ease of access to busy clinicians, with greater flexibility over time and place than a face-to-face or video interview. No members of the participant group were personally known to the authors.

A total of 114 females expressed an interest in taking part. Potential participants were sent an information sheet and consent form (see Supplementary Appendix S1) and were invited for interview if they met eligibility criteria: being a single mother working as a doctor, and having previously left an abusive relationship. No participant currently felt threatened by their ex-partner. Participants were sampled on the basis of mutual availability for phone interview. Verbal consent was taken before starting the in-depth semi-structured telephone interviews. No participant withdrew from the study but one interview was stopped at 16 minutes due to unforeseen circumstances and a mutually convenient time to reschedule could not be found.

The research questions explored issues raised by initial scoping of posts among the online group. The interview guide was developed based on the research questions and previous literature, and adapted as the research developed to ensure pertinent themes raised by prior participants were fully explored (see Supplementary Appendix S2). Interviewees were aware that the interviewer, the first author, was, as stated previously, a member of the online forum and a single parent female doctor. Interviews lasted between 44 and 113 minutes (mean 63 minutes) and were conducted between August 2019 and March 2020. Confidentiality of information was strictly maintained. Interviews were recorded on a digital voice recorder, uploaded to a password-protected computer from the recorder, and then deleted from the recorder. The recordings were transcribed verbatim, checked for accuracy, and anonymised. Recruitment continued until no new themes developed and existing ones were saturated.^15^

An inductive thematic analysis was conducted to identify semantic themes and explore relationships between these. The six phases adapted from the methodology by Braun and Clark were used to facilitate the identification of major themes and enable relationships between the themes to be developed.^16^ NVivo qualitative data management software (version 12) facilitated management of the dataset. Repeated readings of transcripts assisted familiarisation with data and identification of initial codes by the first, third, and last authors, which were then refined through discussion with the second, third, fourth, and last author, all experienced qualitative researchers. The second and last authors are academic GPs; while the third and fourth are non-clinical academics. Transcripts were compared within and between each other using constant comparison,^17^ aiding the iterative search for themes that were then reviewed, defined, and named. As well as using this inductive and iterative approach, the analysis was rooted in a theoretical framework derived from the literature on the female experience of DA,^3^^,^^8^^,^^18^^–^^25^ DA among females from higher socioeconomic groups,^26^ and literature regarding necessity of specialised services for doctors with mental health problems.^27^^–^^30^ Disconfirming cases were sought to further develop the analysis.

An audit trail of study procedures was kept and field notes were made both during and after each interview, which were shared with the team to aid reflexivity and analysis. Efforts were made for the themes to reflect data, rather than preconceived ideas, through reflexivity and discussion between all authors in the multidisciplinary team. All participants read the final draft to ensure they were happy with steps taken to maintain confidentiality and that the researchers had not misinterpreted the participants. There was no disagreement with the results.

## RESULTS

A total of 21 participants aged 32 to 62 years were recruited. Demographical characteristics of the participants are shown in Supplementary Table S1. Most participants defined themselves as ‘White British’ with job roles ranging from GP to consultant, F1/F2 doctor, and registrar. Participants gave accounts of differing forms of abuse ranging from coercive control and threatened physical violence, to actual physical violence and rape. Data were developed into three interrelating themes and subthemes (see Supplementary Figure S1).

### ‘It’s not something that should happen to doctors’

Participants spoke of a sense of failure that their relationship had not worked and they had experienced DA. This was enhanced by feeling that they had not fulfilled their own and society’s expectations of how a doctor should be or what sort of life they should lead. Several participants expressed embarrassment or shame and felt that, as doctors, they *‘should have known better’*:

‘We’re not supposed to be vulnerable; we are supposed to be intelligent, strong women who are not vulnerable. And there is an element of — that I think — you should have known better.’

(Participant [P]9, registrar)

Many expressed that their experiences of DA and subsequent single motherhood had threatened their sense of identity as a doctor. They identified this, as well as geographical relocation for medical jobs, as adding to their social isolation:

‘I feel — very isolated now; I don’t feel like I belong in this group, at all … I’m sort of a bit in no man’s land at the moment. I don’t really belong in one group or the other, you know, they are all married to other doctors and they’re all — at least from the outside — perfect.’

(P9, registrar)

Despite participants feeling shocked that DA had happened to them, in hindsight many identified characteristics of doctors that they felt had made them vulnerable. Many described that being hard working and determined in their professional lives meant they also expected to work hard at their private lives. Many said that they felt they saw the best in people:

‘I think it’s the amount of empathy we have and sympathy for people and — as we discussed earlier as well – going beyond limits to make things work.’

(P19, registrar)

‘So I wonder whether that kind of compassionate kind of person, that is naturally attracted to medicine because you want to fix things and help other people, I think that that probably does make you more at risk … and I think a lot of doctors tend to have those attributes.’

(P16, registrar)

Many participants felt that dealing with ‘bad behaviour’ at work from colleagues, and sometimes patients, had normalised poor treatment at home, which some described as contributing to why they persisted with relationships after they had become abusive:

‘So that just made me think that the culture at work, where we don’t get thanked and we get, quite frankly, bullied quite a lot, just sets you up to think that’s normal at home as well …’

(P3, registrar)

### Difficulties in disclosing and seeking help, particularly for health professionals

Not fitting the stereotype of a female suffering with DA resulted in cues being missed, or disbelief from HPs from whom they sought help as a patient:

*‘The very first time I mentioned it* [to my GP]*, there was a definite, “Oh yes, that wouldn’t happen to you” — probably a lot it was because, yes, I’m a middle-class doctor and that doesn’t happen to middle-class doctors.’*

(P5, GP)

The participant went on to describe how they felt that their GP had closed the conversation down and the participant did not talk further about the abuse. Some felt there was a reticence or embarrassment where other professionals seemed to be discouraging a disclosure:

‘I think — there was — professional courtesy as well, because the health visitor knew there was more going on, that I talked about … and a couple of times the health visitor looked at me and she said, if you say any more, I’m going to need to involve the relevant agencies. So I stopped talking.’

(P8, GP)

Many perceived that their ‘doctor status’ was used negatively against them and some were threatened with being reported to the General Medical Council (GMC):

*‘The first time I ever met a social worker, after my daughter was assaulted* [by the DA perpetrator] *, they turned round and said, “I want your GMC number; I want to see your appraisal.” I don’t know whether it’s specifically because I’m a doctor, but it feels like a witch-hunt. There’s documentation from a social worker in our case notes: “She’s really arrogant, she keeps on telling me she’s a doctor.” I didn’t, but it’s interesting that that is written.*’

(P6, registrar)

Disclosure was also difficult for many because they felt others would view their personal experiences as a reflection that they were inadequate doctors in some way:

‘I think it’s really hard to turn around to people, particularly when you’re a doctor and you’re meant to be the one sitting on the other side of the desk and say, you know, “look at the situation I’ve got myself in.”’

(P4, GP)

‘It’s the social status of the job. You can sometimes think that people will feel that you’re not fit to look after patients when you can’t, you know, you haven’t been able to look after yourself or your family.’

(P2, registrar)

#### When the ex-partner is also a doctor

Participants gave accounts of being labelled mentally ill by their abusive partner as part of the abuse and had concerns regarding professional implications. Where the abusive partner was also a doctor, participants feared that such allegations may have carried more weight both to others, and to themselves:

*‘Less than 48 hours after I left, he was in court. He applied for an order to try and get the children returned to him in* [place]*, saying that, you know, he thought I had postnatal depression and I’d probably become psychotic and that’s why I’d left him.’*

(P4, GP)

‘And he’s a psychiatrist, so — you know — I believed him. He used to tell me that I live in a swirling vortex of despair and sort of made me feel like I’m ... yes, I’m unusual and I believed him … actually I probably was fine and it was all kind of a coercive control kind of thing.’

(P9, registrar)

‘He was always like — “I might have to tell the GMC about you because you’ve got all these problems.”’

(P15, GP)

Where the abusive ex-partner was a doctor, participants felt the ex-partner received better treatment by non-doctor professionals, such as by the Children and Family Court Advisory and Support Service (CAFCASS) because of their doctor status. Having doctor colleagues as character witnesses for the abusive partner could deter the victim-survivor from disclosing the abuse:

‘These were all established GPs and consultants, standing up in court and saying they believe he couldn’t have done any of these things, how great he is and what a wonderful father he is.’

(P4, GP)

#### Lack of confidential services for doctors

Concerns about lack of confidentiality were common. Often the participant knew the team they needed to approach for help and this made disclosure more difficult. Complexity was added where the ex-partner was a doctor locally. Some participants felt the need to protect their abusive partner’s doctor status:

‘He is a doctor and if I had pressed charges then I thought it would just increase the animosity a lot, which wouldn’t be in the best interests of my child again.’

(P18, consultant)

#### Several barriers to seeking help from domestic abuse services

First, some participants spoke about internalised stigma and the difficulties of ‘owning up’ to abuse, and societal externalised stigma where others couldn’t see them as victim-survivors of DA:

‘I got dropped off in a taxi at the domestic abuse centre where I go. And I go and I see some of my patients who are obviously there as well … the taxi driver dropped me off and he said, “Oh, what’s this place?” And I was like — and I said, “it’s — it’s for domestic abuse.” And he said, “Oh, I thought it was a drug — I’ve seen all these waifs and strays going in here.” Even though I wanted to go — “no — that’s not true,” it’s like I still couldn’t … I just kept quiet.’

(P15, GP)

Second, some participants felt that they were not as vulnerable as other victim-survivors of DA because of their financial position and didn’t warrant support from services. Third, many participants were unable to attend DA support services as groups were held during working hours. Most participants did not feel they could ask for time off to attend DA courses, many of which require a weekly commitment. The potential for knowing patients also put some participants off from attending DA services. Other participants did attend, though out of area, but their professional position as doctors caused challenges:

*‘Unfortunately what we do sets us apart and I was put in a really, really difficult position which has led to difficulty because one of these girls* [at a DA support group attended by participant] *was sniffing coke and I had to report it.’*

(P6, registrar)

‘I’ve done the online Freedom Programme but I wouldn’t do it face to face because I live and work in the same area. So it potentially could be patients I know or people that become patients in the future … I could not sit in a room or be a doctor to somebody who’d heard the ins and outs of what I’ve been through.’

(P8, GP)

#### Work can be difficult — though for some it was a source of support

Participants who had taken sick leave while leaving an abusive partner said they felt unsupported on returning to work; but many participants felt unable to take sick leave at all.

Some interviewees described how stress experienced owing to DA had a negative impact on their work:

*‘I never took any time off. I was really stressed. I made lots of mistakes, I had a few complaints and I don’t think they* [colleagues] *really understood how stressed I was.’*

(P5, GP)

Some found that working with victim-survivors of DA was more difficult because they were reminded of their own experiences, especially while their own abuse was recent:

*‘I found it a real struggle to do that job because too many things were hitting too close to home for me. People obviously would disclose domestic violence and I would just shut down and that’s obviously not — you can’t do that … Very early on I told my clinical supervisor at that job, and he was like — it’s a bit early days, let’s just see. And again I tried to muddle through but at the end of 6 months, he gave me a terrible CS* [clinical supervisor] *report as well, saying all these things that, “You’re not engaging in ward rounds; you’re not really talking to patients.”’*

(P10, registrar)

### Perception of a judgemental medical culture

Many participants perceived medical culture to be judgemental towards doctors with problems in their personal lives:

*‘My PTSD* [post-traumatic stress disorder as a result of DA] *is not a secret, but am I going to tell them what happened to me last year, fuck no.* [Interviewer: ‘Why not?’] *... Because the medical profession are judgey bastards.’*

(P6, registrar)

Doctors in training (F1-2 and registrars) felt that compassion needed to be shown when planning rotas and deployment for doctors who lacked childcare support, and that clinical and educational supervisors needed to be more supportive to trainees, especially those experiencing DA who may be more socially isolated:

*‘There was no support in any sense of the word, at all. I had an MSF* [multi-source feedback] *at the time that was quite interesting because it’s one of the only ones I’ve ever had that hasn’t been 100% positive … and no one thought to question why one MSF had gone awry amongst — at that point — a 10-year career.’*

(P6, registrar)

In contrast, a few participants, particularly consultants, felt well supported at work:

*‘I used to leave the house in the morning in tears and walked to work in tears. Honestly, I got to work sometimes and cried to my ODP* [operating department practitioner] *, you know, and they were amazing. And that’s what got me through it.’*

(P14, consultant)

Another consultant reflected on how important it was for patient safety to be working with a team that understood what was going on in their personal life:

*‘So there was a couple of times* [when participant was operating] *where I would say “look, I haven’t had much sleep and if I’m doing something stupid please can you let me know.”’*

(P13, consultant)

After the DA was over and they had recovered from the acute stress, participants felt that their own experiences of DA had enabled them to challenge their own stereotypical views of which females can become victim-survivors, and to show more empathy.

### What helps?

Participants felt that the most helpful things were those that helped to break their sense of isolation. Peer support from the online forum was extremely valuable in helping them understand that they were not alone, as well as inspiring them to take action:

‘Oh, I think it’s absolutely brilliant, absolutely brilliant. It gives so much support – some of the stories you just think, oh my goodness, this is so — unbelievably, you know, terrifying, but also there’s been such a lot of inspiring stuff, of people properly rebuilding their lives.’

(P13, consultant)

Participants often didn’t talk explicitly about DA, but gave information to HPs that led to some HPs probing further, and when they did, participants were grateful. Participants also found it helpful when their GP validated their concerns, expressed sympathy or showed empathy, and treated them as patients:

‘I explained everything to her and she was like, “this is — you know — domestic abuse; this isn’t normal.” And I think — it was like — yes. It was a massive relief, to be honest, because I don’t think I knew what on earth was going on.’

(P16, registrar)

‘The GP was lovely, really, really nice and supportive … She was a nice person, that was an empathetic person who wasn’t acting judgementally towards me, so I think that’s as good as it gets normally, isn’t it?’

(P11, GP)

A few examples were given where a professional had acted as an advocate for participants, which was highly valued by participants. When participants had sought help from DA services, they mainly found it positive, particularly valuing specialist counselling, PTSD support, and support in attending court:

*‘Actually, you know, this specialist support* [from an IDVA]*, if I hadn’t have had it, I’m absolutely sure I would have committed suicide.’*

(P6, registrar)

## DISCUSSION

### Summary

Being a doctor was important to participants’ understanding of their experiences of DA. Responders expressed a sense of shame and embarrassment around disclosing DA, feeling that doctors are expected to achieve a certain lifestyle and to ‘know better’.

The main barrier to accessing support concerned confidentiality, for instance when doctors knew the professional in a personal or professional capacity. Other barriers included perceived stigma, for instance around allegations of mental health problems, particularly where the abusive partner was also a doctor. Participants, particularly those in training, expressed concern that disclosure of DA to their workplace may not be managed sympathetically. They felt that the culture of medicine allowed little time for self-care, and many had felt unable to take sick leave. After the DA was over and they had recovered from the acute stress, participants felt that their own experiences of DA and subsequent single parenthood had enabled them to challenge their own stereotypical views and to show more empathy. Participants reported that breaking their sense of isolation through online peer support and empathetic care from other professionals, often their GP, was most helpful.

In conclusion, DA impacts on the work and wellbeing of female doctors, who face unique barriers to help seeking and reporting DA. The work environment is often experienced as unsupportive, victim-survivors often do not feel able to talk about the abuse confidentially, and they fear consequences of reporting. As a result, they feel socially and professionally isolated. An online peer support group can help to break this sense of isolation, but specialised confidential support services are also required to help doctors experiencing DA.

### Strengths and limitations

Though there have been powerful personal accounts of doctors’ experience of DA,^31^ to the authors’ knowledge this is the first qualitative study exploring their experiences more fully.

Limitations include that participants were recruited from a support group specifically for doctors, and thus these females may self-identify as doctors more than doctor victim-survivors who use other support groups not specifically for doctors. Recruiting through other sources, or interviews being carried out by a non-doctor, may have led to different findings and may be useful for further research. Though demographical data from participants was not obtained in advance of the interviews, a very diverse group was recruited. Interviews focused on participants’ experiences of DA in the context of their role as a doctor and this may have affected the present findings. Some concepts such as ‘professional courtesy’ could have been further explored.

Recruiting doctor victim-survivors who have not yet left the abusive relationship may also have different findings but it may be difficult to recruit such doctors. The study focused on understanding the lived experience of female doctor victim-survivors of DA rather than on developing solutions, but several potential solutions did emerge.

### Comparison with existing literature

The British Medical Association, in their research on the mental health of doctors, found that *‘there is an inequality of access to health and wellbeing services for doctors that must end.’* ^32^ Evidence shows that doctors experience unique barriers to accessing mental health services.^27^^,^^30^ The present findings suggest that barriers to doctors seeking support for DA include stigma and being treated as a doctor rather than a patient, in addition to the barriers faced by other people experiencing DA. Festinger’s ‘social comparison theory’ suggests that people determine their self-worth by comparing themselves to others,^33^ and helps to explain why victim-survivors of DA experienced a loss of identity as a doctor.

Research suggests that being ‘resilient’ can result in victim-survivors tolerating abusive relationships for longer.^3^ Resilience is a quality required by doctors through their long training and exposure to death, distress, and disability.^34^ Participants perceived that this resilience meant they were more likely to remain in an abusive relationship.

Work has been identified as a protective factor for DA victim-survivors.^35^ However, studies show that doctors are more likely to be dissatisfied with their work–life balance compared with the general population, and that bullying is common at medical school and in the medical profession.^34^ There is an urgent need to redress the *‘culture of blame, name, and shame’* in medicine.^34^ The present finding that seeking help for DA can be hard in a working culture where ‘weakness’ is not tolerated reflects the literature on experiences of mental ill health among doctors.^36^

Stereotypes of the typical abused female continue to be held by HPs,^37^ by society, and by victim-survivors themselves. Domestic abuse victim-survivors from higher socioeconomic groups, such as doctors, are often isolated due to not accessing public DA support.^26^ It is important to understand how females recognise abuse in themselves and *‘homogenised assumptions of abuse are structurally created and exclude/stigmatise females whose experiences are “different.”’* ^38^

### Implications for research and practice

A confidential DA service for doctors would allow doctor victim-survivors to access support without risk of meeting their patients and colleagues. Domestic abuse among doctors needs to be acknowledged by NHS workplaces to ensure suitable policies are in place. Greater awareness of existing recommendations among organisations and employers could help support doctor victim-survivors.^39^ A shift away from the culture of presenteeism^40^ in medicine is required to allow doctors experiencing stress at work, or in their personal lives, to be supported to take leave to recover.

Domestic abuse training in medical schools is insufficient.^41^ Training needs to be delivered sensitively, recognising that the problem affects all females, recognising that doctors can also be victims. When females do disclose abuse to clinicians, the response may not always be appropriate.^9^ Healthcare professionals need to feel able to respond to cues around DA or disclosure of DA among people of all backgrounds. Further research is needed among people in other professional roles, in the health service, and beyond to understand whether they face similar barriers to accessing help and support, and to better understand some of the issues that emerged from the present study, such as ‘professional courtesy’.
